# Breaking new ground in family planning communication

**DOI:** 10.9745/GHSP-D-14-00192

**Published:** 2014-12-02

## Abstract

The Urban Reproductive Health Initiative has shown impact on contraceptive use from its communication components even within a few years, as described in 2 GHSP articles. One specifically addressed “ideation” about family planning in detail and was able to show both changes in ideation due to program exposure and correlated changes in contraceptive use. The other used a sophisticated analytical technique that indicated the observed changes in contraceptive use resulted from exposure to the communication efforts, and not just because people more prone to adopt family planning were also more likely to recall exposure to the communication messages.

The Urban Reproductive Health Initiative, funded by the Bill & Melinda Gates Foundation and implemented in 4 countries of Africa and Asia, is breaking new ground in the field of family planning communication. Two articles in this issue of GHSP describe and evaluate the initiative's communication efforts after about 2 years of implementation.

## Endogeneity Addressed Through a Longitudinal Design

The first article, a broader evaluation across all 4 sites led by Speizer of the Measurement, Learning & Evaluation (MLE) project at the University of North Carolina, indeed found impact of exposure to various program efforts (such as recalling a radio program or receiving information at a community event) on contraceptive method use.^1^ However, a perennial problem in such evaluations of communication efforts is concern about “endogeneity”—that is, that people who are more aware or proactive may be more likely *both* to being exposed (or to remember being exposed) to such efforts and also to adopting family planning. Because the longitudinal evaluation design included interviewing many of the same women twice (at baseline and midterm), this endogeneity would have remained fairly constant over time within the same individual. In a sense, women serve as their own controls. By using the technique of comparing 2 complementary statistical approaches, “fixed effects” and “random effects” models, and finding that they gave similar results, the authors conclude such endogeneity was not likely a problem in this situation.

## Changing Ideation to Improve Contraceptive Use

The paper by Krenn and colleagues on the Nigerian Urban Reproductive Health Initiative is notable because they used communication and a client-centered approach as the leading part of the comprehensive family planning program, and they also describe a much deeper and richer dive into the Nigeria program.^2^ The programmers promoted and measured a set of positive “ideational” constructs related to family planning, such as individuals' knowledge and attitudes toward family planning but also their perceptions of the attitudes of key people in their social network, such as peers, partners, and religious leaders, as well as self-efficacy to use contraception. The project had marked impact on a number of these factors, such as women's perception that their peers supported family planning. And positive changes in ideation were associated with increases in contraceptive use.

Beyond these groundbreaking communication efforts, the Urban Reproductive Health Initiative, of course, includes a major “supply side” component as well. The Nigeria program found increases in contraceptive use overall. Thoughtfully adapting to the local context, the program identified a gap in services in slum neighborhoods and instituted a mobile service outreach model to serve those areas. Notably by the end of the reported time frame, such mobile outreach was accounting for about half of the family planning clients served by project-supported clinics. Clearly, both supply and demand are important. We look forward to more findings from this important initiative in the future. —*Global Health: Science and Practice*
